# *Polygonatum sibiricum saponin* Exerts Beneficial Hypoglycemic Effects in Type 2 Diabetes Mice by Improving Hepatic Insulin Resistance and Glycogen Synthesis-Related Proteins

**DOI:** 10.3390/nu14245222

**Published:** 2022-12-08

**Authors:** Zefu Chen, Jiayuan Luo, Mingjie Jia, Yangyang Chai, Yihong Bao

**Affiliations:** 1 School of Forestry, Northeast Forestry University, Harbin 150040, China; 2 Key Laboratory of Forest Food Resources Utilization of Heilongjiang Province, Harbin 150040, China

**Keywords:** *Polygonatum sibiricum*, Saponin, T2DM, hypoglycemic, hepatic insulin resistance, glycogen synthesis, important gut bacteria

## Abstract

Type 2 diabetes mellitus (T2DM) is a systemic metabolic disorder characterized by insulin deficiency and insulin resistance. Recently, it has become a significant threat to public health. *Polygonatum sibiricum saponin* (PSS) has potential hypoglycemic effects, but its specific mechanism needs further study. In this study, PSS significantly decreased the level of blood glucose, water intake, and the organ index in diabetic mice. Meanwhile, PSS effectively reduced the content of total triglyceride (TG), total cholesterol (TCHO), low-density lipoprotein cholesterol (LDL-C), alanine aminotransferase (ALT), and aspartate aminotransferase (AST) in the blood, and increased the content of high-density lipoprotein cholesterol (HDL-C). This suggests that PSS could reduce the content of blood lipids and initially improve the damage of hepatocytes. We found that PSS alleviated hepatic insulin resistance, repaired islet beta cells, and enabled insulin to play its biological role normally. It also improved oral glucose tolerance and abated serum lipopolysaccharide (LPS) and glycosylated hemoglobin (HbA1c) levels in T2DM mice. Furthermore, studies have found that PSS increased the content of phosphorylated protein kinase B (AKT), thereby promoting the effect of glucose transporter 4 (GLUT-4), and activating glycogen synthase kinase 3beta (GSK-3β) and glycogen synthase (GS) proteins to promote hepatic glycogen synthesis. Finally, we found that PSS could promote the growth of beneficial bacteria such as *Bifidobacterium* and *Lactobacillus*, reduce the growth of harmful bacteria such as *Enterococcus* and *Enterobacter*, and preliminarily improve the composition of important bacteria in the intestine. These studies indicate that PSS has an excellent hypoglycemic effect, which provides a potential new treatment for T2DM and guidance for more in-depth research.

## 1. Introduction

Diabetes mellitus is a complex systemic metabolic disease characterized by persistent hyperglycemia [1]. Hyperglycemia is a disease caused by the insufficient secretion of insulin or the insulin’s biological effects being compromised (such as insulin resistance), or a combination of both [2,3]. Although diabetes mellitus is an easily preventable disease, the number of patients suffering from diabetes mellitus is increasing worldwide. It has been recognized by many countries as a serious public safety problem [4,5]. China is the country with the largest number of diabetic patients in the world [6]. According to IDF statistics in 2021 [7], there were 141 million diabetic patients aged 20–79 in China, which is expected to increase to 174 million by 2045. Diabetes mellitus is divided into type 1 diabetes mellitus (T1DM) and type 2 diabetes mellitus (T2DM), of which T2DM is the most common type of diabetes, accounting for more than 90% of the total number of diabetic patients [8,9]. Long-term, persistent high blood sugar can have many adverse effects on the human body, resulting in chronic damage and causing the dysfunction of various tissues, especially in the eyes, kidneys [10], heart [11], blood vessels, and nerves [12], which seriously affect the normal daily life of patients. Therefore, in this context, it is necessary to find an effective treatment for T2DM to inhibit its development.

In order to treat diabetes, pharmaceutical companies and researchers have developed a series of particular drugs for relieving diabetes. However, because T2DM cannot be completely cured, patients develop drug resistance after long-term use, and there are usually side effects due to the drugs [13], such as liver and kidney damage, hypoglycemia, gastrointestinal and digestive problems, etc. [14,15,16]. Since the effects of drug treatments are not very satisfactory, people have turned their attention to diet therapy. Numerous studies have shown that active substances in many edible plants have positive effects with regard to the treatment of diabetes [17,18,19], such as polysaccharides and saponins in plants [20,21,22]. Moreover, these plants are all natural and nontoxic to the human body; therefore, they are ideal raw materials for making natural medicines for treating diabetes.

*Polygonatum sibiricum* is a plant of the Liliaceae family and is widely distributed in China, Korea, Mongolia, and eastern Russia [23]. The rhizome of *Polygonatum sibiricum* is a commonly used traditional Chinese medicine and is the main material of many traditional Chinese medicine formulations [24]. It can treat symptoms such as weakness of the spleen and stomach, physical fatigue, and other symptoms, is thought to have anticancer [25] and antidepressant [26] properties, and is used in the treatment of tuberculosis, ringworm, and so on [27,28]. *Polygonatum sibiricum saponin* (PSS) is one of the main active substances in *Polygonatum sibiricum* [29,30]. PSS has the function of improving learning and memory; has antitumor, antioxidant, and anti-free radical properties; protects the ischemic myocardium; and regulates immunity [31]. Therefore, there is a great demand for PSS. Previous studies have found that saponins in plants have hypoglycemic effects. Zhu et al. found that ginsenosides can alleviate insulin resistance by regulating the Sirt1/pGC-1α signaling pathway [22]. Elekofehinti et al. found that saponins from *Solanum anguivi Lam.* fruits achieve antidiabetic effects by attenuating hyperglycemia-mediated oxidative stress and hyperlipidemia [32]. Currently, studies have preliminarily proved that PSS can also reduce blood glucose [33], but the specific mechanisms in vivo have not been revealed. More scientific evidence is needed to prove the actual effect of PSS on diabetes treatment. This is of great practical significance for the development of new natural medicines for the treatment of diabetes.

The purpose of this study was to reveal the specific hypoglycemic effect of PSS in vivo. A mice model of T2DM induced by a combination of high-fat diet feeding (HFD) and streptozotocin (STZ) was used for the study.

## 2. Materials and Methods

### 2.1. Materials

PSS was extracted by using the methods presented in the literature [33]. Total triglyceride (TG), total cholesterol (TCHO), high-density lipoprotein cholesterol (HDL-C), low-density lipoprotein cholesterol (LDL-C), alanine aminotransferase (ALT), and aspartate aminotransferase (AST) kits were purchased from the Nanjing Jiancheng Bioengineering Research Institute (Nanjing, China). ELISA kits for insulin, serum lipopolysaccharide (LPS), and glycosylated hemoglobin (HbA1c) levels were purchased from Shanghai Enzyme Linked Biotechnology Co., Ltd. (Shanghai, China). Blood glucose meters and blood glucose test strips were purchased from Wuding Biotechnology Co., Ltd. (Taiwan, China). STZ was purchased from Sigma Chemical Co. (St. Louis, MO, USA).

### 2.2. Animals

Four-week-old Kunming mice, weighing about 18–25 g, were ordered from Jinan Pengyue Experimental Animal Breeding Co., Ltd. (Jinan, China) (animal license number: SCXK (Lu) 2019-0003). All the mice were male and bred indoors. The temperature was maintained at 24 ± 3 °C, the relative humidity was maintained at 50 ± 10%, the light and dark times were maintained for 12 h each, and ventilation was maintained.

### 2.3. Experimental Design

After adaptive culture, 40 mice were fed with the HFD, and the other 8 mice were fed with regular rodent food. After one month of the HFD, STZ was used for intraperitoneal injection modeling. The mice were fasted for 12 h before injection, and each mouse was injected intraperitoneally with an injection amount of 60 mg/kg for two consecutive days. The normal group was injected with the same amount of normal saline. Three days later, the mice were fasted for 12 h, and then the blood glucose of each mouse was measured using a blood glucose meter. A fasting blood glucose level ≥11.1 mmol/L was considered a successful model and could be included in the experiment.

The successfully modeled mice were divided into the T2DM control (TC) group, high-dose control (HDC) group, middle-dose control (MDC) group, low-dose control (LDC) group, and positive control (PC) group, and the mice that did not participate in the modeling made up the normal control (NC) group, with 8 mice in each group (*n* = 8). The HDC, MDC, and LDC groups were intragastrically administered with PSS. The PC group was intragastrically administered with metformin. The NC and TC groups were intragastrically administered with the same amount of normal saline. The amount of the intragastric administration of PSS extract was 1600 mg/kg in the HDC group, 800 mg/kg in the MDC group, and 400 mg/kg in the LDC group. The amount of the intragastric administration of metformin (concentration of 20 mg/mL) in the PC group was 250 mg/kg. All the mice were gavaged once a day for four consecutive weeks. Body weight, fasting blood glucose, and food and water consumption were recorded weekly. Afterward, all the mice were sacrificed under general anesthesia with ether (≥99%). Then, the experimental materials were collected and stored until further testing. All the animal experiments were approved by the Ethics Committee of Experimental Animal Research at Northeast Forestry University.

### 2.4. Oral Glucose Tolerance Test (OGTT) and Insulin Tolerance Test (ITT)

The oral glucose tolerance test (OGTT) was performed to investigate the glucose tolerance of mice in different groups. Specifically, after the mice were fasted for 12 h, the basal blood glucose was measured by taking tail vein blood. Then, each mouse was gavaged with 2 g/kg body weight of glucose solution, and the state of the mouse was observed. The tail vein blood was collected from mice at 30, 60, 90, and 120 min, and the blood was used to measure the blood glucose of the mice via a glucometer. Blood glucose values were recorded at these time points, OGTT curves were made, and the area under the curve (AUC) was calculated.

The insulin tolerance test (ITT) was also performed to examine the insulin resistance of mice in different groups. After fasting for 12 h, the test was carried out. The venous blood of the mice’s tails was taken and the blood glucose was measured with a blood glucose meter, which was used as the basic blood glucose. Each mouse was injected with 1 IU/kg body weight of human insulin using an intraperitoneal injection method. Tail vein blood was collected from mice at 30, 60, 90, and 120 min and used to measure the blood glucose. An ITT curve was made from the blood glucose values and the area under the curve (AUC) was calculated.

### 2.5. Analysis of Serum Lipid Levels

The commercial kit from the Nanjing Jiancheng Bioengineering Research Institute (Nanjing, China) was used to detect the contents of TCHO, TG, HDL-C, and LDL-C in the mouse serum. Every operational step was strictly performed following the manufacturer’s instructions. The atherosclerosis index (AI) was calculated as (TCHO-HDL-C)/HDL-C.

### 2.6. Determination of Blood Glucose and Insulin Levels

Blood was collected from the tail vein of the mice, and the blood glucose of the mice was measured using a blood glucose meter. The mouse serum insulin was determined using an ELISA kit (Shanghai Enzyme Linked Biotechnology Co., Ltd., Shanghai, China), and all operations were performed strictly following the manufacturer’s instructions. The homeostasis model assessment of the insulin resistance (HOMA-IR) calculation formula is HOMA-IR = [fasting blood glucose (mmol/L) × fasting plasma insulin (mIU)/L)]/22.5 and the homeostasis model assessment of the insulin sensitivity (HOMA-β) calculation formula is HOMA-β = 20 × fasting plasma insulin (mIU/L)/fasting blood glucose (mmol/L) −3.5.

### 2.7. Determination of Serum Lipopolysaccharide (LPS) and Glycosylated Hemoglobin (HbA1c) Levels

The LPS and HbA1c levels of mice were determined by using an ELISA kit (Shanghai Enzyme Linked Biotechnology Co., Ltd., Shanghai, China). All the operations were carried out in strict accordance with the manufacturer’s instructions.

### 2.8. Determination of Liver Glycogen Levels

The content of glycogen in the mouse liver was measured using a commercial kit from Nanjing Jiancheng Bioengineering Research Institute (Nanjing, China). It was used according to the manufacturer’s instructions. Briefly, livers were removed from the −80 °C freezer to thaw and were weighed, then homogenized with 3 volumes of lye and heated together in a boiling water bath for 20 min for hydrolysis. The hydrolyzed solution was mixed with distilled water to prepare a liver glycogen detection solution with a concentration of 1%. Then, the content of the liver glycogen is obtained by colorimetric determination.

### 2.9. Western Blot

The expression of four proteins, phosphorylated protein kinase B (p-AKT), glucose transporter 4 (GLUT-4), glycogen synthase kinase 3 beta (GSK-3β), and glycogen synthase (GS), was detected using Western blot. The liver tissue was removed from the −80 °C freezer and thawed. After weighing, the liver tissue was added to a RIPA lysis buffer (strong) containing 1 mM phenylmethylsulfonyl fluoride (PMSF) and 1% protease inhibitors and phosphatase inhibitors. The tissue was frozen, homogenized, and lysed using liquid nitrogen. The protein concentration in the liver grinding fluid was measured using a BCA protein quantification kit (Bioss, Beijing, China). The concentration of proteins was 18 mg/mL. The proteins in the liver were separated by SDS-PAGE electrophoresis and then transferred to immunoblotting PVDF membranes, and the membranes were blocked with 5% skimmed milk for 2 h. After blocking, they were combined with rabbit anti-p-AKT (1:1000), rabbit anti-GLUT-4 (1:1000), mouse anti-GSK-3β (1:1000), and rabbit anti-GS (1:1000) at 4 °C. After overnight incubation, the membranes were washed with TBS-Tween 20 (TBST). Membranes were incubated with an HRP-conjugated rabbit antibody (1:20,000) or mouse antibody (1:20,000). The resulting immune complexes were developed with an ultrasensitive ECL luminescence kit (Shanghai Biyuntian Biotechnology Co., Ltd., Shanghai, China) and visualized using a gel imaging system (Tanon 5200, Shanghai, China).

### 2.10. Plate Colony Counting

The feces of mice were mixed with the PBS solution at a mass ratio of 1:9 to prepare a fecal bacterial suspension with a concentration of 10^−1^. The diluted fecal bacterial suspension was obtained by successively diluting in a gradient.

There were four kinds of bacteria-specific culture media, each equipped with either *Bifidobacterium*, *Lactobacillus*, *Enterococcus*, or *Enterobacter*. A sterile pipette was used to draw 0.1 mL of bacterial solutions of different concentrations to inoculate the medium (three replicates for each dilution). Then, a sterile spreader was used to spread the bacterial solution evenly on the plate. The smeared plate was placed on the table, a layer of medium was poured to seal it, the plate was inverted to cultivate in a constant-temperature incubator (37 °C, 48 h), and the colonies were counted after they grew.

### 2.11. Statistical Analysis

All data are presented as mean ± standard deviation (SD). A one-way analysis of variance (ANOVA) was performed on all statistical data using SPSS software (Version 22.0, Armonk, NY, USA), and *p* < 0.05 was considered statistically significant. All reported values were calculated independently three times.

## 3. Results

### 3.1. Effects of PSS on General Physiological Indices

The changes in body weight, food intake, and water intake of each group were monitored weekly during the experimental period. The food intake results of each group are shown in Table 1. It can be seen that the food intake of diabetic animals was significantly higher than that of the NC group mice, indicating that diabetic symptoms increased the food intake of mice. After treatment with PSS and metformin, the final food intake of each group decreased, but there was no significant change. The results of water intake are shown in Table 2. The water intake of diabetic mice was significantly higher than that of the NC group mice. This was because diabetes can increase the concentration of blood glucose, and the body’s regulation of blood glucose causes increased urination and water deficiency. Therefore, the water intake of diabetic mice was significantly increased. After the intervention with PSS and metformin, the water intake of the HDC, MDC, LDC, and PC groups decreased, indicating that the symptoms of diabetes were alleviated. Increased food intake and water intake are considered typical symptoms of diabetes. The experimental results showed that PSS could reduce the food and water intake in diabetic mice; therefore, the unhealthy state of diabetic mice can be alleviated.

**Figure 1:**
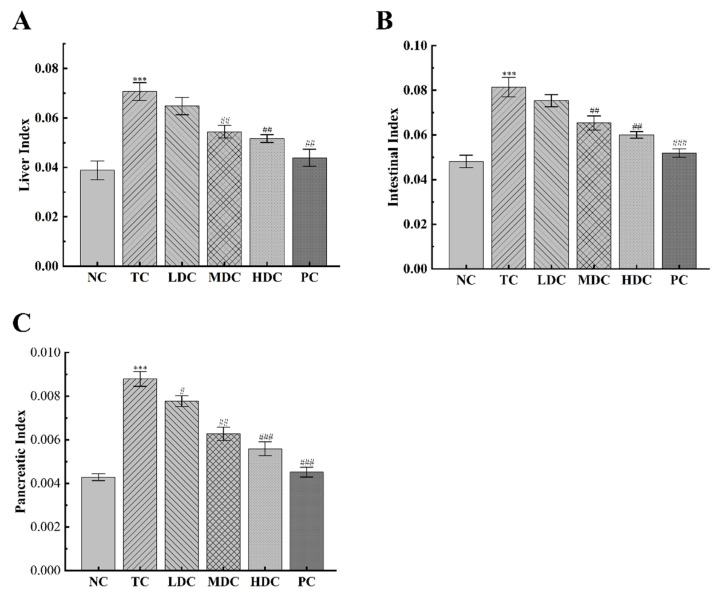
Effect of PSS on organ index. (**A**) The liver index. (**B**) The intestinal index. (**C**) The pancreatic index. The organ index was calculated by dividing the organ weight by the body weight. Data are presented as mean ± SD (n = 8); *** *p* < 0.001 compared to the NC group; ^#^ *p* < 0.05, ^##^ *p* < 0.01, and ^###^ *p* < 0.001 compared to the TC group.

The body weight changes of each group are recorded in Table 3. The initial body weight of the mice in each group was not significantly different. However, the weight of the diabetic mice was slightly higher than that of the NC group mice, which was because the high-sugar and high-fat diet feeding caused obesity in diabetic mice. Mice in all groups showed an upward trend in body weight over the four-week period, as the four-week period allowed mice to grow and, thus, gain weight. The increase in the organ index is also one of the typical symptoms of diabetes. As can be seen in Figure 1A–C, the liver index, intestinal index, and pancreatic index of diabetic mice were increased by 82.06%, 69.21%, and 104.87%, respectively, compared to the NC group, indicating that organs were damaged. The treatment with PSS relieved this symptom. In the LDC, MDC, HDC, and PC groups, the liver index decreased by 8.35%, 23.02%, 26.93%, and 39.33%, respectively, compared to the TC group. The intestinal index decreased by 7.49%, 19.71%, 27.53%, and 36.25%. Additionally, the pancreatic index was reduced by 11.58%, 28.57%, 36.42%, and 48.61%.

### 3.2. Effects of PSS on Serum Lipids and Biochemical Indices of Hepatocytes

**Figure 2:**
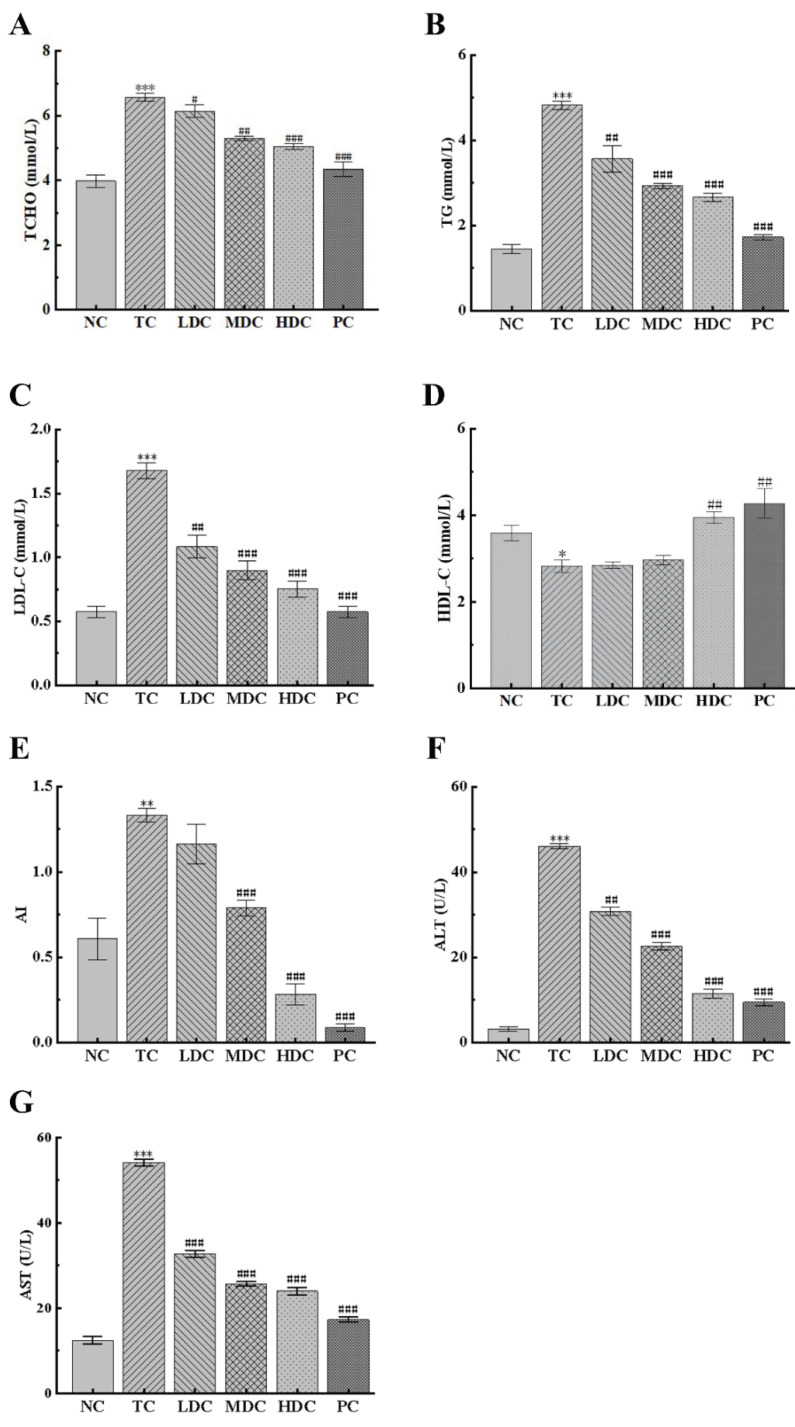
Effect of PSS on general biochemical indicators. The content of (**A**) total cholesterol (TCHO), (**B**) total triglyceride (TG), (**C**) low-density lipoprotein cholesterol (LDL-C), (**D**) high-density lipoprotein cholesterol (HDL-C), (**F**) alanine aminotransferase (ALT), and (**G**) aspartate aminotransferase (AST) in the serum of mice. (**E**) The atherosclerosis index (AI). Data are presented as mean ± SD (n = 8); * *p* < 0.05, ** *p* < 0.01, and *** *p* < 0.001 compared to the NC group; ^#^ *p* < 0.05, ^##^ *p* < 0.01, and ^###^ *p* < 0.001 compared to the TC group.

In order to understand the effect of diabetes on blood lipids and the hypolipidemic effect of PSS, the contents of TCHO, TG, LDL-C, and HDL-C in the sera of mice were detected. As can be seen from Figure 2A,B, the contents of TCHO and TG in the TC group were significantly increased by 65.08% and 233.21%, respectively, compared to the NC group. This was caused by obesity and an abnormal lipid metabolism. The treatment with PSS and metformin for four weeks reduced the contents of TCHO and TG in the body. Compared with the TC group, the MDC group, HDC group, and PC group had a significantly reduction in TCHO and TG content, bringing the blood lipids back to normal.

LDL-C can carry cholesterol into cells, which can easily cause atherosclerosis. It can be seen from Figure 2C that the LDL-C content of mice in the TC group was significantly higher than that in the NC group, and there was a high risk of atherosclerosis. PSS and metformin can reduce the LDL-C content in the body. Compared with the TC group, the LDL-C levels in the blood were significantly reduced by 35.47%, 46.51%, 55.23%, and 65.69% in the LDC, MDC, HDC, and PC groups, respectively. This slowed down the transport of cholesterol and reduced the risk of atherosclerosis. The same conclusion can be drawn from the results of AI (Figure 2E). HDL-C can promote the metabolism of cholesterol. As can be seen from Figure 2D, the HDL-C content of the TC group was reduced by 23.41% compared to the NC group. Additionally, compared with the TC group, the HDL-C content in the HDC and PC groups increased by 40.04% and 51.48%, respectively. This indicates that the PSS and metformin can promote the decomposition of cholesterol and show an excellent therapeutic effect.

The contents of ALT and AST in the serum can indicate whether hepatocytes are damaged. Thus, we determined the contents of ALT and AST in the serum of each group of mice (Figure 2F,G). The contents of ALT and AST in the TC group were significantly higher than those in the NC group, indicating that the liver cells were damaged. The contents of ALT and AST in the blood of mice in the four treatment groups were significantly reduced, suggesting that PSS had a positive effect in repairing liver damage, and the treatment effect of the high and medium doses was the best.

### 3.3. Effect of PSS on Glucose Tolerance in Mice

**Figure 3:**
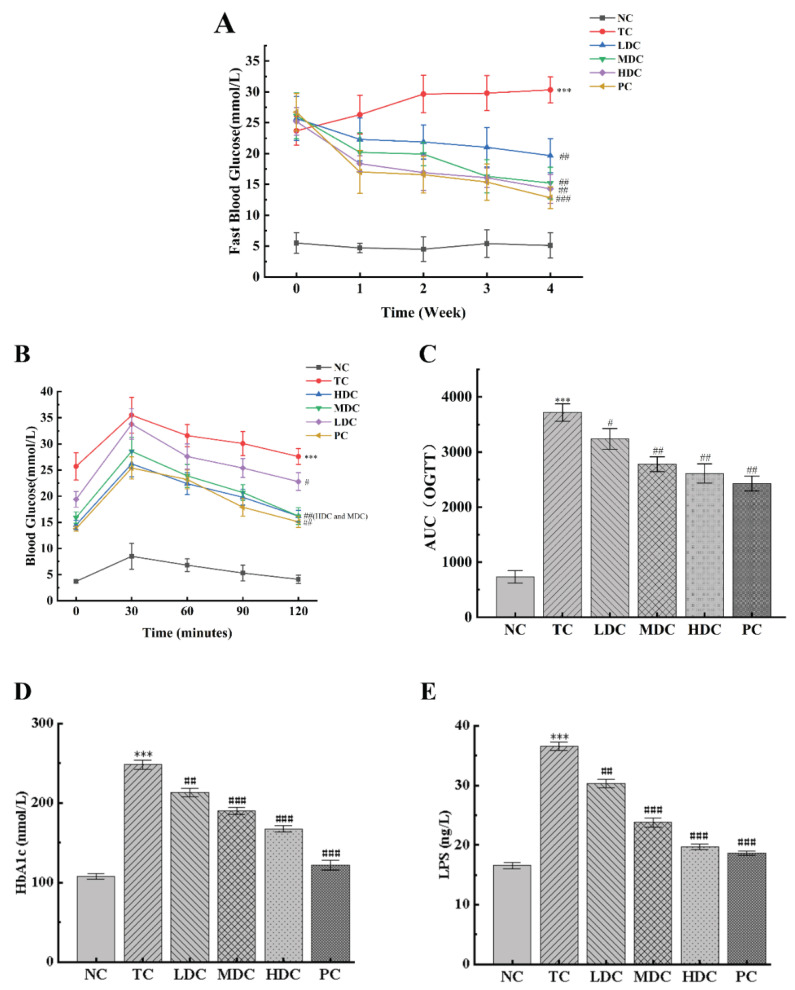
Effect of PSS on glucose tolerance in mice. (**A**) Fasting blood glucose. (**B**) Oral glucose tolerance test (OGTT) experiment results. (**C**) The area under the curve (AUC) of OGTT. The contents of (**D**) glycosylated hemoglobin (HbA1c). The contents of (**E**) serum lipopolysaccharides (LPS). Data are presented as mean ± SD (n = 8); *** *p* < 0.001 compared to the NC group; ^#^ *p* < 0.05, ^##^ *p* < 0.01, and ^###^ *p* < 0.001 compared to the TC group.

During the four-week experiment, the changes in the fasting blood glucose in mice were recorded weekly (Figure 3A). It can be seen from the changes in the blood glucose values that the blood glucose values of mice in the NC group fluctuated within the four weeks, but they all fluctuated around the value of 5, and the blood glucose value was at the normal level. The blood glucose levels of mice in the TC group continued to increase from week 0 and reached a peak at week 4, maintaining a high level. The blood glucose values in the HDC group, MDC group, LDC group, and PC group during week 0 also showed hyperglycemia symptoms; however, the blood glucose values slowly decreased to 14.28 mmol/L, 15.22 mmol/L, 19.67 mmol/L, and 12.84 mmol/L after 4 weeks of treatment with PSS and metformin, respectively, which were significantly different from those in the TC group (*p* < 0.01), and which alleviated the symptoms of hyperglycemia.

The results of the OGTT are shown in Figure 3B. In the experimental time of 120 min, the blood glucose values of all the groups reached the peak value at 30 min, and generally, showed a trend of first increasing and then decreasing. Compared with the NC group, the blood glucose levels of the mice in the TC group were significantly higher (*p* < 0.001), indicating that the glucose tolerance of the mice in the TC group was severely impaired by diabetes. In the NC group, the blood glucose levels recovered to the initial level after oral glucose administration for 120 min. Compared with the TC group, the blood glucose levels of the MDC group, HDC group, and PC group were significantly lower (*p* < 0.01), and they all recovered to the initial level after 120 min of administration. Similar conclusions were obtained by calculating the AUC results (Figure 3C). The AUC_OGTT_ of the TC group was significantly higher than that of the NC group (*p* < 0.001), and the AUC_OGTT_ values of the LDC group, MDC group, HDC group, and PC group were all lower than those of the TC group, which decreased by 12.87%, 25.21%, 29.69%, and 34.61%, respectively. Among them, the high and middle doses of PSS had a more significant effect on the OGTT results of diabetic mice.

Figure 3D shows that the HbA1c levels in the TC group were significantly higher than that in the NC group by 130.68% (*p* < 0.001). After treatment with PSS and metformin, the HbA1c levels decreased (*p* < 0.01), of which the LDC, MDC, HDC, and PC groups decreased by 14.22%, 23.48%, 32.59%, and 50.95%, respectively, compared to the TC group. We also measured the LPS content in the serum of mice (Figure 3E). The results showed that the LPS content in the TC group was significantly higher than that in the NC group (*p* < 0.001). After treatment with PSS and metformin, the LPS content of mice in each group decreased significantly (*p* < 0.01). Among them, the MDC, HDC, and PC groups showing favorable treatment effects, with decreases of 34.89%, 46.14%, and 49.07%, respectively.

### 3.4. PSS Increases Insulin Sensitivity in Diabetic Mice

**Figure 4:**
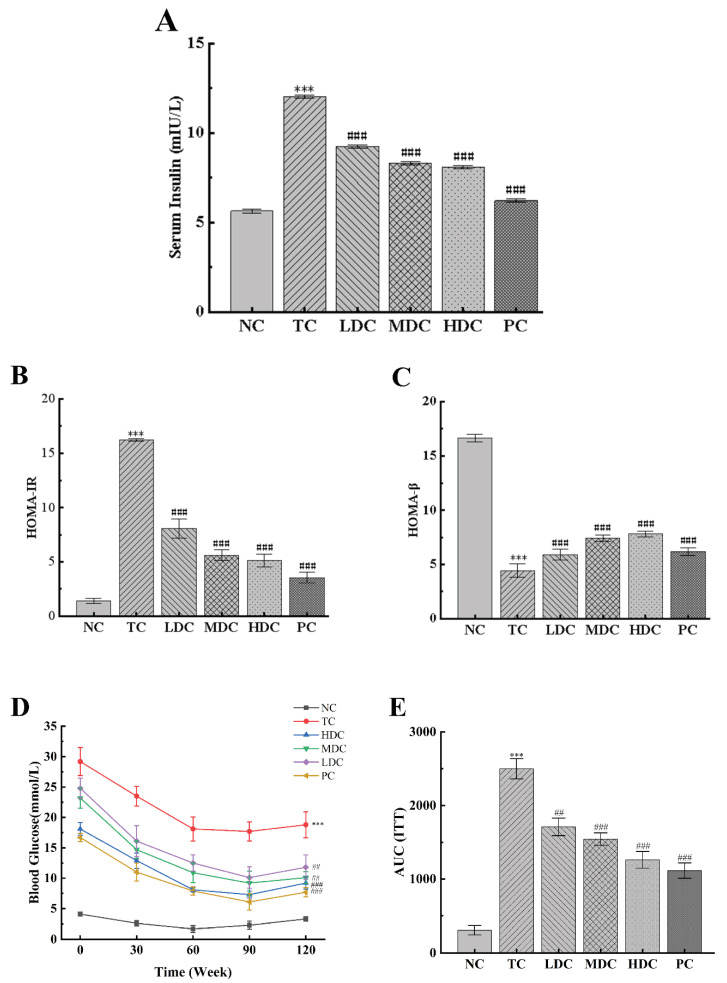
Effect of PSS on insulin sensitivity in mice. (**A**) The serum insulin content. (**B**) The HOMA-IR scores. (**C**) The HOMA-β scores. (**D**) Insulin tolerance test (ITT) experiment results. (**E**) The area under the curve (AUC) of ITT. Data are presented as mean ± SD (n = 8); *** *p* < 0.001 compared to the NC group; ^##^ *p* < 0.01 and ^###^ *p* < 0.001 compared to the TC group.

We measured the effect of PSS and metformin on the insulin content in mice (Figure 4A). The insulin content in the TC group was significantly higher than that in the NC group. This is due to the development of insulin resistance, which makes it difficult for insulin to work. After treatment with metformin and PSS, the insulin content of mice in the LDC group, MDC group, HDC group, and PC group decreased (*p* < 0.001), of which the MDC group, HDC group, and PC group showed a positive therapeutic effect. HOMA-IR and HOMA-β indices were calculated according to blood glucose and insulin levels (Figure 4B,C). It was observed that the HOMA-IR index increased and the HOMA-β index decreased in the TC group compared to the NC group. After treatment with PSS and metformin, the HOMA-IR index of the LDC group, MDC group, HDC group, and PC group decreased by 50.22%, 65.43%, 68.39%, and 78.13%, respectively, compared with the TC group, indicating that the symptoms of insulin resistance in diabetic mice were improved. Compared with the TC group, the HOMA-β index increased by 32.66%, 66.89%, 76.13%, and 39.64%, indicating that PSS could treat the damage to islet β cells caused by diabetes and restore the function of islet β cells. Among them, the middle and high doses of PSS showed a positive effect.

The ITT experiments were performed three days before the end of the experiment (Figure 4D,E). It can be observed that the blood glucose levels of the mice in all groups began to decrease after insulin injection, showing a trend of first decreasing and then increasing as a whole. Within 120 min of the experiment, the blood glucose of mice in the NC group was significantly lower than that in the TC group. The blood glucose of mice in the NC group returned to the baseline level after 120 min of insulin injection. The blood glucose levels of diabetic mice treated with PSS and metformin were significantly lower than those of untreated diabetic mice (*p* < 0.01). Similar trends can be shown by computing the AUC of the ITT: The AUC_ITT_ of the TC group was significantly higher than that of the NC group (*p* < 0.001), and the AUC_ITT_ of the LDC group, MDC group, HDC group, and PC group was significantly lower than that of the TC group (*p* < 0.01), indicating that the diabetic mice treated with PSS and metformin were more sensitive to insulin. These results suggest that PSS could improve the insulin sensitivity of diabetic mice and repair damaged islet β cells.

### 3.5. Effects of PSS on Insulin Pathway-Related Proteins and Hepatic Glycogen Synthesis-Related Proteins

**Figure 5:**
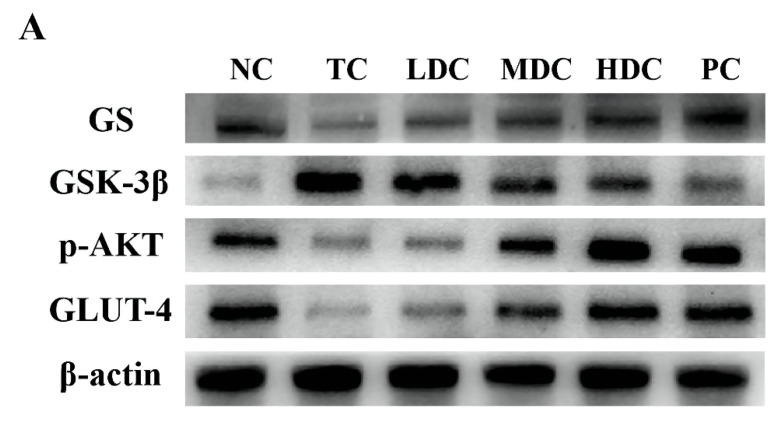

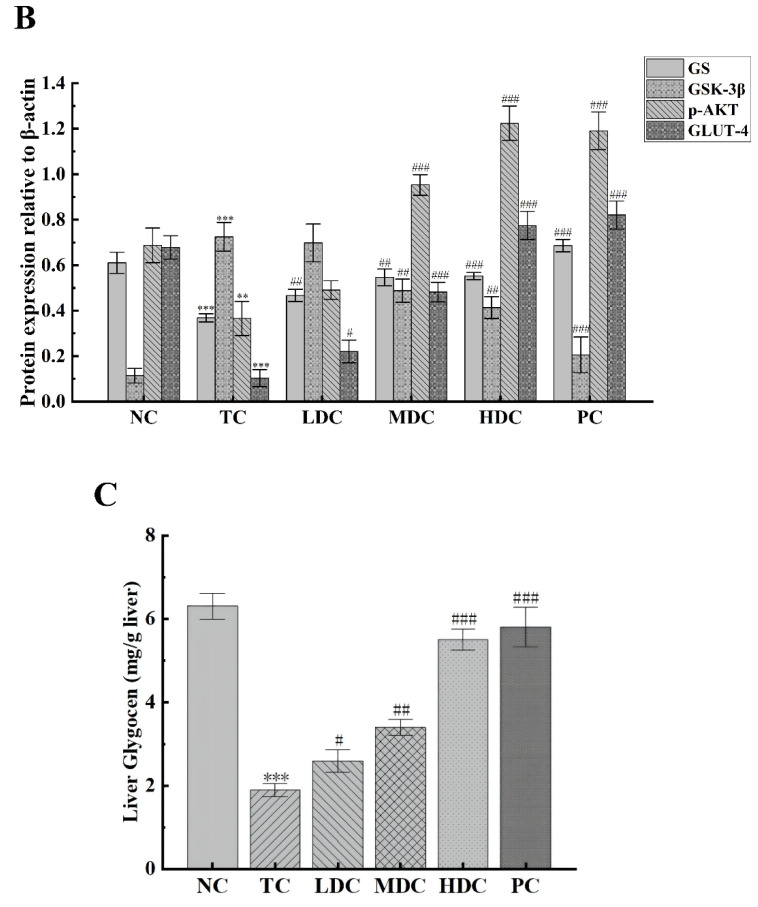
(**A**) The protein expression levels of glycogen synthase (GS), glycogen synthase kinase 3beta (GSK-3β), phosphorylated protein kinase B (p-AKT), and glucose transporter 4 (GLUT-4) were determined via Western blot. (**B**) Protein levels of the above proteins were quantified by densitometry, all of which were normalized to β-actin. (**C**) Liver glycogen content. Data are presented as mean ± SD (n = 8); ** *p* < 0.01 and *** *p* < 0.001 compared to the NC group; ^#^ *p* < 0.05, ^##^ *p* < 0.01, and ^###^ *p* < 0.001 compared to the TC group.

In order to study the effect of PSS on the expression of p-AKT and its downstream proteins in the mice liver, the related proteins in the liver were detected by using the Western blot method. p-AKT is phosphorylated AKT, which is the main form of AKT. GLUT-4 is responsible for transporting the glucose in the blood into cells for decomposition and utilization to maintain the blood glucose balance. These two proteins are essential in the human body, and thus, we examined the expression of p-AKT and GLUT-4 proteins in mice (Figure 5A,B). The contents of p-AKT and GLUT-4 in the TC group were significantly lower than those in the NC group (*p* < 0.01). Diabetes inhibits the production of p-AKT and the activation of GLUT-4, thereby reducing the catabolism of glucose in the body and resulting in increased blood glucose. The contents of p-AKT and GLUT-4 in diabetic mice were increased after treatment with PSS and metformin, and the catabolism of glucose in vivo was restored. Among them, the middle and high doses of PSS showed a good recovery effect.

To explore the effect of PSS on hepatic glycogen synthesis-related proteins in mice, we detected the expression levels of GS and GSK-3β in mice (Figure 5A,B). Compared with the NC group, the expression of GS in the TC group was significantly decreased, but the content of GS in the diabetic mice was increased after the treatment with PSS. The level of GSK-3β in the TC group was significantly higher than that in the NC group, which inhibited the synthesis of liver glycogen in vivo. PSS alleviated the overexpression of GSK-3β in diabetic mice. Compared with the TC group, the content of GSK-3β in the MDC group, HDC group, and PC group significantly decreased (*p* < 0.01), but there was no significant change in the LDC group. The results for the liver glycogen content in mice also showed the same trend (Figure 5C). The content of liver glycogen in mice in the TC group was significantly lower than that in the NC group (*p* < 0.001). The treatment of PSS restored the synthesis of liver glycogen. It significantly increased the liver glycogen content in the body, and out of all the PSS treatments, the high dose of PSS was the most effective at restoring the synthesis of liver glycogen in the body. These data suggest that diabetes impairs liver glycogen synthesis in mice, which PSS ameliorates.

### 3.6. Effect of PSS on Important Intestinal Bacteria

**Figure 6:**
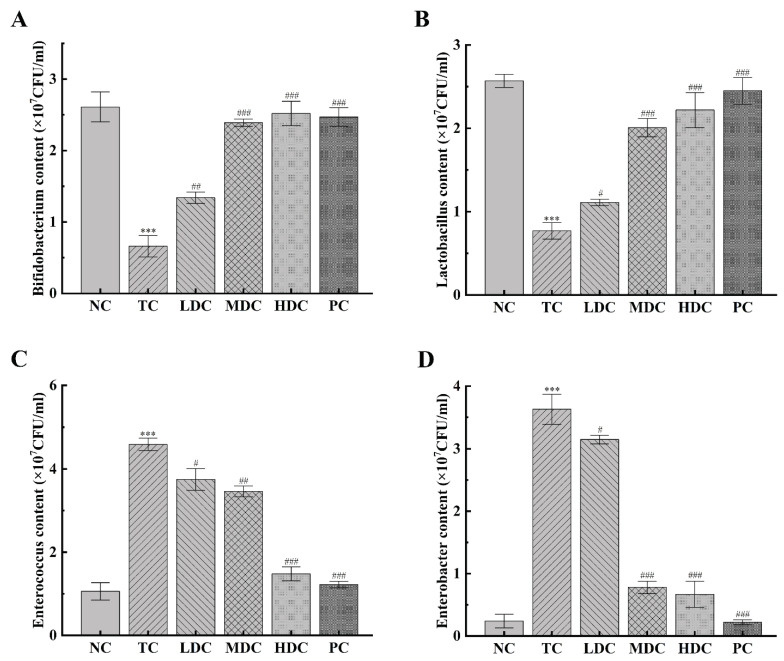
The effect of PSS on the number of important bacteria in the gut was investigated. The number of (**A**) Bifidobacterium, (**B**) Lactobacillus, (**C**) Enterococcus, and (**D**) Enterobacter in the intestinal tract of mice was detected. Data are presented as mean ± SD (n = 8); *** *p* < 0.001 compared to the NC group; ^#^ *p* < 0.05, ^##^ *p* < 0.01, and ^###^ *p* < 0.001 compared to the TC group.

In order to understand the effect of PSS on the number of *Bifidobacterium*, *Lactobacillus*, *Enterococcus*, and *Enterobacter* in the intestine of mice, we detected the changes in the number of these four bacteria (Figure 6A–D). *Bifidobacterium* and *Lactobacillus* are beneficial bacteria in the gut. *Bifidobacterium* is a kind of biological barrier and has many essential physiological functions in the human body [34,35], and *Lactobacillus* can promote the absorption of nutrients [36]. The number of these two bacteria was significantly decreased in the TC group compared to the NC group (*p* < 0.001), by 74.71% and 70.16%, respectively. This indicates that the beneficial bacteria in the gut were destroyed and the number decreased. The contents of these two bacteria in the intestinal tract of diabetic mice were increased after the treatment with PSS. However, the low dose of PSS had little effect on the recovery of these two bacteria (*p* < 0.05), and the best therapeutic effect was from the middle dose and the high dose of PSS (*p* < 0.001). *Enterococcus* and *Enterobacter* are harmful bacteria in the gut. *Enterococcus* easily causes a variety of infections [37], and *Enterobacter* can cause digestive tract diseases [38,39]. The contents of *Enterococcus* and *Enterobacter* in the TC group were significantly higher than those in the NC group (*p* < 0.001), which increased by 3.33 times and 14.12 times, respectively, and therefore, had a higher risk of pathogenicity. Diabetic mice treated with high doses of PSS and metformin for four weeks significantly experienced a decrease in the number of these two bacteria in the intestinal tract (*p* < 0.001), thereby reducing the risk of disease. The above results show that the high dose of PSS greatly impacts the recovery of the number of beneficial bacteria and the reduction in the number of harmful bacteria, suggesting that PSS could improve the flora structure in the intestinal tract of diabetic mice.

## 4. Discussion

T2DM is a chronic metabolic disease whose most apparent symptom is persistent hyperglycemia in the body. Long-term hyperglycemia will cause a series of abnormal symptoms in the body [40,41,42]. *Polygonatum sibiricum* contains a variety of bioactive ingredients and has become a hot research topic. In recent years, some studies have found that PSS has an antihyperglycemic effect, and we have conducted in-depth research on this in the past.

Within four weeks, we monitored the changes in the body weight, food intake, water intake, and blood glucose levels of mice. The results showed that PSS could significantly reduce the fasting blood glucose levels, which were proportional to the dose. Increased water intake and food intake were seen as typical symptoms of diabetes [43,44]. After treatment with PSS, the food intake and water intake of treated mice were lower than those of diabetic mice, indicating that these symptoms were alleviated. Since the diabetic mice that were fed a high-fat diet developed symptoms of obesity, the body weight was higher than that of the normal mice, and the weight continued to increase over the 4 weeks. The results of the organ index also showed the same trend. After feeding the mice the PSS, the trend of weight gain slowed down, and the organ index decreased, indicating that the symptoms of obesity were initially improved.

Dyslipidemia is a major symptom of T2DM [45]. Glucose and lipid metabolism in the human body are interrelated in many aspects. When the glucose metabolism in the body is abnormal, the lipid metabolism will also be damaged. Therefore, T2DM patients also often experience dyslipidemia symptoms, which easily cause hyperlipidemia [18]. We studied the blood lipid levels in T2DM mice, including the TCHO, TG, HDL-C, and LDL-C contents. The results showed that the blood lipid levels of T2DM mice were significantly decreased after treatment with PSS, and the increase in HDL-C can also prove this trend. HDL-C can transport cholesterol and promote the decomposition of cholesterol into bile acids for metabolism. Increased HDL-C levels will promote cholesterol catabolism, decrease blood lipid levels, and reduce the risk of hyperlipidemia. The liver is an important organ for lipid metabolism and glucose metabolism. When mice have dyslipidemia, it may be due to damage to the liver, accompanied by symptoms such as hepatic insulin resistance and impaired glycogen synthesis [46]. ALT and AST are the most sensitive indicators for liver cell damage. Our study found that AST and ALT levels in T2DM mice significantly increased, indicating that liver cells were severely damaged. Therefore, the injury of liver cells may be one of the crucial reasons for the increase in blood glucose and dyslipidemia in the body. The treatment of PSS alleviated this phenomenon, suggesting that PSS may promote the progress of glucose metabolism and lipid metabolism by repairing liver cells, thereby exerting the effects of reducing blood lipids and lowering blood glucose.

Insulin resistance is a negative physiological state in which the body or cells are not sensitive to insulin, and it is difficult for insulin to be functional. This symptom exists throughout the progression of T2DM. The insulin levels in T2DM mice are significantly higher than in normal mice. This persistent hyperinsulinemic state could damage the body, exacerbate symptoms of insulin resistance, and destroy insulin tolerance. From the HOMA-IR index and HOMA-β index, it can be seen that the insulin resistance symptoms of T2DM mice were severe, and the islet β cells were damaged [47]. After treatment with PSS, the level of the HOMA-IR index was decreased and the level of the HOMA-β index was increased, indicating that the symptoms of insulin resistance were relieved and the islet β cells were repaired. The results of the ITT experiments also showed that PSS could effectively improve the insulin sensitivity of T2DM mice. Increasing the body’s sensitivity to insulin could make insulin work better and promote the metabolism and utilization of glucose. In the OGTT experiment, we also found that PSS improved the symptoms of glucose tolerance in T2DM mice, and HbA1c and LPS contents in vivo were also reduced [48]. HbA1c can reflect the average blood glucose level over the past few weeks. LPS are the main components of the cell wall of Gram-negative bacteria. They easily induce the production of inflammatory factors and insulin resistance in the body after the cell ruptures and releases into the blood. In previous studies, it has been demonstrated that a reduction in plasma LPS levels is associated with improved symptoms of diabetes and obesity. These results suggest that PSS could reduce blood glucose by changing insulin resistance, insulin tolerance, and glucose tolerance.

It is worth mentioning that, in addition to improving insulin resistance, PSS also increased the expression of p-AKT in the liver. p-AKT is a key factor that can regulate insulin activity throughout the body and control the expression of GLUT-4 protein and other critical downstream proteins. At the same time, p-AKT can also activate the downstream GS and GSK-3β proteins to promote the synthesis of liver glycogen, which plays a crucial role in the treatment of T2DM [43]. In our study, we found that the administration of PSS to T2DM mice could partially restore the normal operation of the insulin signaling pathway, such as increased AKT phosphorylation and GLUT-4 expression. Therefore, it can be speculated that PSS may help insulin perform its normal physiological function by repairing the insulin pathway. The restoration of the insulin signaling pathway can alleviate the symptoms of insulin resistance, restore the utilization of insulin by cells, and increase insulin activity. Furthermore, the increased expression of GLUT-4 can promote the transport of glucose in cells and accelerate the decomposition of glucose [49]. PSS could promote the synthesis of liver glycogen by increasing the expression of GS and decreasing the expression of GSK-3β in vivo. The increase in p-AKT content will also stimulate the GS and GSK-3β proteins to play a role. The combined effect of these two actions could make the body use glucose to synthesize liver glycogen vigorously, accelerating the utilization of glucose in the body and lowering the blood glucose level. This suggests that PSS may affect the whole hepatic glycogen synthesis pathway.

The change in intestinal flora is considered to be one of the critical factors for glucose and lipid metabolism disorders in the occurrence of T2DM. The host’s dietary composition and eating habits will significantly affect the composition of intestinal microflora as the intestinal flora structure is significantly affected by many active ingredients in food [50,51]. This suggests that a food intervention to change the intestinal flora composition to treat T2DM may be an effective strategy. We found significant changes in the number of some representative bacteria, such as *Bifidobacterium*, *Lactobacillus*, *Enterococcus*, and *Enterobacter*, in the gut of T2DM mice before and after feeding with PSS. PSS can be highly fermented and metabolized by the intestinal flora in the intestine, which can promote the growth of some beneficial intestinal bacteria and enable the beneficial intestinal bacteria to use the PSS to produce short-chain fatty acids, thereby improving the intestinal microbial environment [52]. As a kind of beneficial physiological bacteria, *Bifidobacterium* has many critical physiological functions such as being a biological barrier and having an immune enhancement effect [53,54]. In terms of nutritional physiology, *Lactobacillus* can improve the body’s digestion and absorption of nutrients such as protein, lactose, and calcium, and can produce a variety of vitamins [55,56]. It can also inhibit the reproduction of pathogenic bacteria in the intestinal tract and regulate immunity [57]. The numbers of these two kinds of bacteria increased significantly after T2DM mice were fed with PSS, which promoted the health of the body to increase. *Enterococcus* and *Enterobacter* are prone to cause urinary tract infections and skin and soft tissue infections, as well as life-threatening abdominal infections, sepsis, pericarditis, and meningitis [58,59]. In addition, they are difficult to treat after infection [60,61]. PSS reduced the number of *Enterococcus* and *Enterobacter* in T2DM mice, and reduced the risk of pathogenicity and infection. These results suggest that PSS may have the ability to improve the intestinal microbial environment and change the composition of intestinal flora, thereby having a positive impact on the treatment of T2DM. Considering that PSS promotes the growth of *Bifidobacterium* and *Lactobacillus* in vivo, we speculate that PSS may have a probiotic effect, which proves that PSS has great potential for protecting human health.

In summary, our study found that PSS had a noticeable hypoglycemic effect. Additionally, we newly discovered that PSS can increase cellular glucose transport and alleviate insulin resistance by restoring the expression of p-AKT and its downstream GLUT-4 protein. At the same time, it can preliminarily improve the amount of some important bacteria in the intestine, which has a positive effect on the health regulation of the body. Based on these findings, we speculate that PSS may play a beneficial role in T2DM by restoring the insulin pathway and improving intestinal flora. However, more research is needed to prove this speculation.

## 5. Conclusions

In conclusion, this study demonstrated that PSS has a significant hypoglycemic effect on STZ-induced diabetic mice. Taking PSS can reduce blood lipids, and improve glucose tolerance and insulin resistance. Furthermore, the impaired expression of p-AKT and its downstream GLUT-4 protein can also be repaired by PSS while promoting hepatic glycogen synthesis. High doses of PSS can preliminarily improve the number of some important bacteria in the intestinal tract of diabetic mice. Taken together, our study found that PSS can play an antidiabetic role through these above aspects, and PSS can be used as a natural plant active ingredient for the treatment of T2DM. The more specific hypoglycemic mechanism of PSS in vivo deserves further study.

## Figures and Tables

**Table 1:** Food intake of mice in each group (g/mouse/day).

Group	1 Week	2 Week	3 Week	4 Week
NC	5.53 ± 0.27	5.39 ± 0.87	6.01 ± 0.68	5.18 ± 0.36
TC	11.71 ± 0.67 ***	11.97 ± 1.58 ***	12.59 ± 1.66 ***	13.12 ± 0.93 ***
HDC	10.93 ± 1.71	11.11 ± 0.79	10.62 ± 1.58 ^#^	9.83 ± 1.27 ^#^
MDC	11.64 ± 0.43	11.72 ± 0.23	10.98 ± 1.12	9.92 ± 1.01 ^#^
LDC	12.03 ± 0.94	12.27 ± 0.86	11.85 ± 0.40	11.62 ± 0.71
PC	11.50 ± 0.51	10.93 ± 1.31	11.12 ± 0.27	10.14 ± 0.43 ^#^

NC—normal control group; TC—T2DM control group; HDC—high-dose control group; MDC—middle-dose control group; LDC—low-dose control group; PC—positive control group. Data are presented as mean ± SD (n = 8); *** *p* < 0.001 compared to the NC group; ^#^ *p* < 0.05 compared to the TC group.

**Table 2:** Water intake of mice in each group (mL/mouse/day).

Group	1 Week	2 Week	3 Week	4 Week
NC	5.82 ± 0.71	5.29 ± 0.80	6.39 ± 1.11	5.65 ± 1.03
TC	38.72 ± 2.36 ***	40.88 ± 1.99 ***	42.48 ± 3.32 ***	46.19 ± 2.85 ***
HDC	38.07 ± 1.61	33.93 ± 2.55 ^#^	30.93 ± 1.78 ^##^	29.36 ± 1.13 ^##^
MDC	39.60 ± 1.42	37.01 ± 1.98	35.82 ± 1.23 ^#^	32.79 ± 0.78 ^##^
LDC	40.06 ± 2.03	39.81 ± 1.59	38.87 ± 0.41	36.71 ± 2.24 ^#^
PC	36.78 ± 0.51	31.07 ± 1.41 ^#^	28.11 ± 1.04 ^##^	23.68 ± 1.35 ^##^

NC—normal control group; TC—T2DM control group; HDC—high-dose control group; MDC—middle-dose control group; LDC—low-dose control group; PC—positive control group. Data are presented as mean ± SD (n = 8); *** *p* < 0.001 compared to the NC group; ^#^ *p* < 0.05 and ^##^ *p* < 0.01 compared to the TC group.

**Table 3:** Body weight of mice in each group (g).

Group	0 Week	1 Week	2 Week	3 Week	4 Week
NC	32.98 ± 3.54	33.82 ± 2.99	35.01 ± 2.46	35.62 ± 3.13	36.68 ± 3.29
TC	38.90 ± 4.67	40.08 ± 5.23	44.45 ± 4.62 *	47.18 ± 2.91 *	49.08 ± 3.04 *
HDC	36.47 ± 3.07	37.41 ± 2.41	38.26 ± 1.70	39.13 ± 2.76	39.49 ± 3.35 ^#^
MDC	37.71 ± 2.39	38.22 ± 1.40	39.60 ± 2.26	40.13 ± 3.22	40.56 ± 2.73 ^#^
LDC	37.77 ± 2.76	38.93 ± 4.21	40.01 ± 2.73	40.51 ± 3.44	40.88 ± 3.85 ^#^
PC	36.84 ± 3.17	37.53 ± 3.24	38.28 ± 3.76	38.92 ± 4.77 ^#^	39.51 ± 3.69 ^#^

NC—normal control group; TC—T2DM control group; HDC—high-dose control group; MDC—middle-dose control group; LDC—low-dose control group; PC—positive control group. Data are presented as mean ± SD (n = 8); * *p* < 0.05 compared to the NC group; ^#^ *p* < 0.05 compared to the TC group.

## Data Availability

Not applicable.
